# The Mechanism of Anterior Cruciate Ligament Injuries in the National Football League: A Systematic Video Review

**DOI:** 10.7759/cureus.34291

**Published:** 2023-01-27

**Authors:** Samuel Schick, Colin K Cantrell, Bradley Young, Zachary Mosher, Michael Ewing, Joseph W Elphingstone, Eugene Brabston, Brent A Ponce, Amit M Momaya

**Affiliations:** 1 Department of Internal Medicine, West Virginia University, Morgantown, USA; 2 Department of Orthopaedic Surgery, Northwestern University Feinberg School of Medicine, Chicago, USA; 3 Department of Orthopaedic Surgery, Atrium Health, Charlotte, USA; 4 Department of Orthopaedic Surgery, Campbell Clinic, Memphis, USA; 5 Department of Orthopaedic Surgery, University of Missouri School of Medicine, Columbia, USA; 6 Department of Orthopaedic Surgery, University of Alabama at Birmingham School of Medicine, Birmingham, USA; 7 Department of Orthopaedic Surgery, Hughston Clinic, Columbus, USA

**Keywords:** risk factor, american football, nfl, video analysis, acl injury

## Abstract

Introduction

Anterior cruciate ligament (ACL) injuries are common among American football athletes, although few studies have performed video analyses of ACL injuries to better understand the injury mechanism. This work aims to characterize the mechanism of ACL injury during professional football competitions using video analysis. We hypothesize that football-specific injury trends will emerge, including high rates of contact injuries and associations with shallow knee and hip flexion angles (0°-30°).

Methods

Videos of professional football players suffering ACL injuries from 2007 to 2016 were analyzed. Injured players were identified using the injured reserve (IR) lists of the National Football League (NFL), and videos were discovered via a systematic Google search. Descriptive statistics and frequency analyses were performed on all variables using the Statistical Package for the Social Sciences (SPSS) version 23.0 (IBM SPSS Statistics, Armonk, NY, USA).

Results

Of the 429 ACL injuries identified, 53 (12%) videos were available. Deceleration was the most common injury maneuver, present in 32 (60%) athletes. Thirty-one (58%) players suffered contact injuries. Twenty-eight (53%) injuries demonstrated valgus collapse of the knee, and 26 (49%) had neutral knee rotation. Defensive backs (26%) and wide receivers (23%) were the most frequently injured positions.

Conclusion

Overall, we found that most ACL injuries had preceding contact, deceleration, shallow hip and knee flexion, and heel strike, and subsequent valgus collapse and neutral knee rotation. This understanding of American football-specific ACL tear mechanisms could help direct the focus of future injury prevention training modalities.

## Introduction

Anterior cruciate ligament (ACL) tears are devastating injuries in professional athletes, affecting approximately 4% of players each year. ACL injuries are particularly impactful on the careers of athletes in the National Football League (NFL), with studies demonstrating a return to play rate of 63%-82% and a salary decrease of $2 million over the following four seasons when compared to their uninjured counterparts [1-3]. It has been shown that these injuries are associated with many different movements, such as pivoting, cutting, jumping, accelerating, and decelerating [4,5]. Understanding the risk factors and mechanisms correlated with ACL injuries is important in injury prevention [6].

Recent studies have performed video analyses of basketball, European football (soccer), netball, alpine skiing, Australian football, rugby, and American football players suffering ACL injuries with the goal of highlighting trends in injury mechanism and knee biomechanics before and after injury [7-11]. These studies found that in non-contact sports, the three predominant forms of injury mechanism were landing from jumping, decelerating, and cutting maneuvers. Valgus collapse was also frequently found following injury [11]. Johnston et al. performed a similar study investigating ACL injuries in professional football players. However, despite football being a contact sport, the authors noted that most ACL injuries were non-contact and associated with lateral movement and knee flexion of 0°-45° [12].

In this study, we sought to perform a similar video analysis of ACL injuries in NFL players, with the aim of determining the predominant ACL injury mechanisms in American football. Additionally, we aimed to describe the kinematics immediately before and after ACL injury to better understand the forces directed upon the knee causing injury. We hypothesized that most injuries would be contact-related and occur during abrupt changes in stride and that athletes would be positioned in shallow knee and hip flexion (0°-30°).

## Materials and methods

NFL injured reserve (IR) lists from 2007 to 2016 were reviewed (https://www.foxsports.com/nfl/transactions) (access date: September 25, 2017). Any player whose injury description included the words “leg,” “knee,” or “ACL” was further investigated to determine whether an ACL injury occurred. Once ACL injury was confirmed via injury reports, player profiles/biographies, or press releases, videos of the injuries were accessed via a systematic Google search using the keywords “Player Name” and “ACL Injury Video.” The first five pages of results were viewed. There was no minimum number of video angles required; however, most videos provided more than one for injury analysis. Videos were qualified for review if all data points were visualized. Player demographics such as height, weight, position, age, and race were obtained from NFL.com player rosters.

Three authors independently reviewed and analyzed the videos. The collected data points included the following: horizontal running speed, vertical running speed, acceleration/deceleration/constant speed, player maneuver, player contact (direct/indirect), foot-to-ground contact, knee flexion angle, hip flexion angle, ankle dorsiflexion/plantar flexion, direction of knee collapse, and direction of rotation upon collapse. Joint flexion angles were stratified into 30° intervals to accurately categorize flexion angles for analysis. Additional definitions of injury-associated characteristics can be found in Table 1.

**Table 1:** Definitions and Categories of Injury Characteristics

Horizontal Running Speed		Vertical Running Speed		
0 - No horizontal movement		0 - No jumping or vertical movement		
1 - Slow jog or movement		1 - Slight jump/hop		
2 - Moderate running		2 - Full jump/vertical leap		
3 - Full Sprint				
Player Maneuvers				
Sidestep: player movement in the contralateral direction of the injured knee				
Crossover: player movement toward the ipsilateral side of the injured knee				
Landing: player landing from a vertical jump, leap, or hop				
Stopping: player quickly decelerating to a stop within one step				
Land and step: player lands and steps with the contralateral foot such as in regular running				
Knee Flexion Angles	Hip Flexion Angles		Ankle Flexion Direction and Angles	
0°-30°	0°-30°		Plantar Flexion	Dorsiflexion
30°-60°	30°-60°		0°-30°	0°-30°
60°-90°	60°-90°		30°-60°	30°-60°
90°-120°	90°-120°		60°-90°	60°-90°
Direction of Knee Collapse	Direction of Knee Rotation		Change in Rate of speed	
Neutral	Neutral		No change	
Valgus	Internal		Acceleration	
Varus	External		Deceleration	

Acceleration, deceleration, or constant speed was determined in the time immediately preceding injury. Player contact was determined if there was any contact from another player at the time of injury. Direct contact was defined as contact directly to the injured knee. Indirect contact was defined as any contact to the body other than the injured knee. Foot-to-ground contact (toe versus heel strike) was determined by which portion of the foot struck the ground first at or immediately prior to injury. Data were combined, and all discrepancies were settled via a collective agreement between the reviewers. The authors’ agreement equaled 96% for collected data points before reconciliation.

Descriptive statistics were performed using the Statistical Package for the Social Sciences (SPSS) version 23.0 (IBM SPSS Statistics, Armonk, NY, USA). Frequencies of all categorical variables were gathered. Additionally, descriptive statistics were performed for all continuous variables.

## Results

Over the year period, 429 ACL injuries were identified via the NFL’s IR lists. Of these injuries, publicly accessible videos were available for 53 (12%). The average age for injured players was 27±3 years. Defensive backs and wide receivers sustained the most injuries, with 14 (26%) and 12 (23%), respectively. Forty-four (83%) were first-time ACL injuries, while nine (17%) had experienced a previous injury. Complete player demographics are available in Table 2.

**Table 2:** Demographic Information of Injured Players lbs, pounds; %, percent of representation within the grouping Height is in feet (') and inches ('').

Position		Age (Years)		Height	
Defensive back	14 (26%)	Range	22-35	5’8” to 5’11”	16 (30%)
Receiver/tight end	12 (23%)	Average	26.9	6’0” to 6’3”	30 (57%)
Linebacker	9 (17%)	Median	27	6’4” to 6’7”	7 (13%)
Running back	9 (17%)	Weight (lbs)		Previous ACL Injury	
Quarterback	6 (11%)	180-210	23 (43%)	No	44 (83%)
Defensive line	1 (2%)	211-240	16 (30%)	Yes	9 (17%)
Offensive line	1 (2%)	241-270	12 (23%)	Same knee	6 (67%)
Kicker	1 (2%)	270+	2 (4%)	Contralateral knee	3 (33%)
Race		Knee Injured			
African American	40 (75%)	Right	23 (43%)		
Caucasian	13 (25%)	Left	30 (57%)		

Over half, 31 (58%) players, sustained contact during injury, with 20 (65%) of these sustaining direct contact to the knee and 11 (35%) with indirect contact (shoulders/upper extremities only). Heel strike was associated with 32 (60%) injuries, which was the most common foot position for ground contact. Twenty-six (49%) injuries occurred while the player was moving at a moderate running pace, and 32 (60%) occurred with deceleration. Lastly, only 11 (21%) injuries occurred with leaping/landing from a vertical leap. Complete situational injury statistics can be found in Table 3.

**Table 3:** Athlete Positioning Injury Characteristics Horizontal Running Speed: 0 - no horizontal movement, 1 - slow jog or movement, 2 - moderate running, 3 - full Sprint; Vertical Running Speed: 0 - no jumping or vertical movement, 1 - slight jump/hop, 2 - full jump/vertical leap Flexion of 0° denotes a fully extended leg.

Horizontal Running Speed (Number (%))		Vertical Running Speed (Number (%))		Acceleration/Deceleration/Constant Speed (Number (%))		Foot Strike (Number (%))	
0	6 (11.32%)	0	48 (90.57%)	Acceleration	6 (11.32%)	Heel	32 (60.38%)
1	18 (33.96%)	1	1 (1.89%)	Deceleration	32 (60.38%)	Toeing	21 (39.62%)
2	26 (49.06%)	2	4 (7.55%)	Constant	15 (28.30%)		
3	3 (5.66%)						
Player Maneuvers		Direction of Knee Collapse		Direction of Knee Rotation			
Sidestep	13 (24.53%)	Neutral	20 (37.74%)	Neutral		26 (49.06%)	
Crossover	2 (3.77%)	Valgus	28 (52.83%)	Internal		12 (22.64%)	
Landing	11 (20.75%)	Varus	5 (9.43%)	External		15 (28.30%)	
Stopping	3 (5.66%)						
Land and step	24 (45.28%)						
Knee Flexion Angles		Hip Flexion Angles		Ankle Flexion Direction and Angles			
0°-30°	33 (62.26%)	0°-30°	26 (49.06%)	Plantar Flexion (58.49%)		Dorsiflexion (41.51%)	
30°-60°	9 (16.98%)	30°-60°	19 (35.85%)	0°-30°	28 (52.83%)	0°-30°	21 (39.62%)
60°-90°	9 (16.98%)	60°-90°	8 (15.09%)	30°-60°	3 (5.66%)	30°-60°	0 (0%)
90°-120°	2 (3.77%)	90°-120°	0 (0%)	60°-90°	0 (0%)	60°-90°	1 (1.89%)

Shallow flexion was the most common joint positioning observed among injured athletes. Thirty-three (62%) injuries occurred at 0°-30° knee flexion, 26 (49%) were at 0°-30° hip flexion, and 28 (53%) were at 0°-30° of plantar flexion. The predominant rotation pattern was neutral with 26 (49%) injuries, while valgus directional collapse was noted in 28 (53%) injuries. Full results for lower extremity joint flexion angles, collapse, and rotation upon collapse can be found in Table 3.

Out of the nine athletes with a prior knee injury, six (66%) experienced an ACL injury on the previously repaired knee, four of which were associated with contact (two direct and two indirect). Five (83%) players were decelerating during injury, and four (67%) were landing when injured. Initial heel and toe strikes were even at three (50%) each, and five injuries (83%) occurred with plantar flexion of the ankle and half (3) with mild knee and hip flexion.

## Discussion

This study aimed to use video analysis to assess trends in the ACL injury mechanisms of American football players. The kinematics most commonly encountered with ACL injuries were deceleration, mild knee flexion (0°-30°), and initial foot-to-ground contact through heel strike. Additionally, most injuries were associated with contact from another athlete, either directly to the knee or indirectly to other parts of the body.

Similar to previous soccer literature, most players we analyzed were decelerating at the time of injury [13]. However, this is discordant with Johnston et al., who previously noted that only 10% of ACL injuries in professional American football players were associated with deceleration [12]. Unfortunately, the majority of video analyses of sports-related injuries do not assess this crucial injury characteristic. Numerous studies have examined the forces placed on the ACL during a single-leg landing during various actions and confirmed that increased tension is placed on the ACL during deceleration [14-16]. Several hypotheses have been proposed to explain this biomechanical correlation. One theory purports that deceleration places substantial stress on the ACL in an effort to prevent anterior tibial translation [17]. Another asserts that eccentric quadriceps contraction, as with deceleration, increases the compressive vector within the knee, effectively lowering the injury threshold [18]. The interaction of thigh and lower leg muscle activation likely also plays a role in ACL injuries, especially in non-contact mechanisms [19]. Further research is necessary to illuminate the interplay of biomechanical forces generated during deceleration on the ACL.

Another factor we correlated with ACL injury, consistent with previous literature, was decreased knee flexion [8,13]. Biomechanical studies have demonstrated that the greatest degree of strain is placed on the ACL, relative to other knee ligaments, at low angles of knee flexion (~15°) [20]. When the degree of flexion increases, the ACL is off-loaded and greater stress is placed on other knee ligaments [21]. A similar study to that herein found mild knee flexion, defined as 0°-45°, in 82% of ACL injuries in NFL athletes [12]. The high rate of ACL injury at knee flexion between 0° and 30° (62.3%) observed in our study further supports the previous video analysis and biomechanical studies [7-9,12,13].

This is the first study to associate foot positioning preceding ACL injury in NFL athletes, as most studies on sports-related ACL injuries do not assess or highlight initial foot-to-ground contact as a risk factor for injury [7-9,11-13]. However, a study of rugby players did reveal similar findings with an increased risk of ACL injuries in players that contact the ground with their heels [10]. Kinematic and biomechanical studies supported these results, indicating that initial heel strike decreases the dampening forces of the ankle, thereby transmitting greater forces to the knee [22,23]. Additionally, vibratory forces transmitted to soft tissue compartments around the knee continue to increase with heel strike, as opposed to plateau with toe striking [24]. The compounded forces of heel striking may transmit to the ACL preferentially and increase injury susceptibility.

In contrast to a recent video analysis study of ACL tears in NFL players, our study found that the majority of ACL injuries occurred via a contact mechanism (58%), including both direct and indirect contact. Johnston et al. recorded contact mechanisms in only 27.5% of cases; however, 68% of injuries in their study that were classified as non-contact involved indirect contact [12]. The group considered a contact mechanism only for those injuries in which there was direct contact with the injured extremity, although they did discuss indirect “perturbations” as contributing to injuries [12]. We find this noteworthy due to the fact that distractions, such as blind-sided contact, can alter body motion by impacting muscle activation timing and thereby influence ACL injury risk [23]. Upper limb motion, such as catching a ball, stiff-arming a defender, or throwing a pass, has been demonstrated to increase the potential for ACL injury [25]. Indirect contacts during these actions frequently promote unanticipated reactionary movements by the athlete, such as lateral trunk abduction, which are highly associated with ACL injury [26].

The high injury rates among receivers and defensive backs in our study are likely multifactorial. These positions are engaged in the most running, especially at high speeds, with sharp directional and velocity changes. Such activities have been shown to place increased strain on the ACL and thereby elevate injury risk [5,6,15]. These positions also frequently collide with other athletes during critical plays. As we and other studies have noted, more than 50% of ACL injuries in physical sports, such as rugby and American football, have a contact component [10]. This rate is considerably greater than what is documented in sports such as basketball (28%) and soccer (36%) [7-9]. Additionally, sport-specific movements can influence ACL injury frequency. For example, Krosshaug et al. found that 59% of professional basketball players who suffered an ACL tear did so after landing on their feet from a vertical leap [7]. The same injury pattern was only observed in 21% of our cases. However, NFL athletes do not jump as frequently as they accelerate and change direction, although receivers and defensive backs do often leap for catches and pass deflections. Thus, differences in activity-associated mechanisms between sports make generalizing ACL injury risk associations very difficult. Lastly, from a non-biomechanical perspective, injuries may be seen at a higher frequency in the aforementioned positions because they are most likely to be captured on camera, as technicians focus on ball movement during games. As a result, injuries from linemen or other defensive players after a pass has been made may go undetected on video and therefore be underrepresented in studies.

Given the high prevalence of ACL and other ligamentous injuries, prevention strategies should be a major focus of research. However, there is an absence of literature on the subject in regard to American football athletes. Most ACL injury prevention training studies have been conducted with soccer athletes. One such landmark study, Prevent Knee Injury and Enhance Performance (PEP Program), investigated neuromuscular approaches centered on proprioception exercises and stretching techniques that focus on proper landing technique, engaging knee and hip flexion on landing and lateral maneuvers, avoiding excessive genu valgum on landing and squatting, increasing core, hamstring, gluteus medius, and hip abductor strength, and addressing proper deceleration techniques. Over the two-year trial period, the group of 1,041 high school female soccer players receiving this training revealed an 88% decrease in anterior cruciate ligament injury when compared to age- and skill-matched controls in the first year and a 74% reduction during the second year [27]. Although this study population is not representative of NFL athletes, the principles utilized could be redirected toward American football-specific drills to serve as a powerful tool in reducing ACL injuries. As such, there is a definite gap in the literature that needs to be addressed; the results from injury prevention training programs on American football, along with other contact sports, may not only reduce short-term ligamentous injury risk but also pay dividends by reducing long-term joint damage from post-traumatic osteoarthritis [28].

Limitations

This study is not without limitations. Only a small percentage of ACL injuries that occurred among NFL players (12%) had associated videos available for analysis. Injury rates among the various positions may be influenced by which positions are in the camera’s spotlight, as videographers focus on ball movement during plays. Since we only used publicly available videos and did not use any paid subscription service or obtain videos from individual NFL teams, the percentage of athletes studied was lower than the study conducted by Johnston et al. Further access to such videos would likely alter our results. Also, these videos were produced for fan viewership and not for kinematic analysis. Despite this, there was high agreement between the video data collectors. Given that we performed a descriptive study, a detailed causation analysis could not be performed. This is due to the fact that video analysis alone cannot be used to assess ACL forces generated by various movements.

## Conclusions

Most ACL injuries in NFL athletes are associated with contact, brisk deceleration, and shallow knee and hip flexion angles. Additionally, heel strike is a major kinematic risk factor for injury in this player population. Given the heterogeneity of movements between sports, these conclusions cannot be applied broadly across sports, but an awareness of such associated activities should guide preventative training methods and research to lower ligamentous injuries in athletes.
